# Evaluation of detection probabilities at the water-filtering and initial PCR steps in environmental DNA metabarcoding using a multispecies site occupancy model

**DOI:** 10.1038/s41598-019-40233-1

**Published:** 2019-03-05

**Authors:** Hideyuki Doi, Keiichi Fukaya, Shin-ichiro Oka, Keiichi Sato, Michio Kondoh, Masaki Miya

**Affiliations:** 1 Graduate School of Simulation Studies, University of Hyogo, Minatojima-minamimachi, Kobe, 650-0047 Japan; 2 National Institute for Environmental Studies, Tsukuba, Ibaraki, 305-8506 Japan; 3 The Institute of Statistical Mathematics, Midoricho, Tachikawa, Tokyo, 190-8562 Japan; 4 Okinawa Churashima Research Center, Ishikawa, Motobu, Okinawa, 905-0206 Japan; 5 Faculty of Science and Technology, Ryukoku University, Seta-Oe, Otsu, 520-2194 Shiga, Japan; 6 Graduate School of Life Sciences Tohoku University 6-3 Aramaki Aza Aoba, Aoba-ku, Sendai, 980-8578 Japan; 7 Department of Ecology and Environmental Sciences, Natural History Museum and Institute, Aoba-cho, Chuo-ku, Chiba, 260-8682 Japan

## Abstract

Environmental DNA (eDNA) metabarcoding is a recently developed method to assess biodiversity based on a high-throughput parallel DNA sequencing applied to DNA present in the ecosystem. Although eDNA metabarcoding enables a rapid assessment of biodiversity, it is prone to species detection errors that may occur at sequential steps in field sampling, laboratory experiments, and bioinformatics. In this study, we illustrate how the error rates in the eDNA metabarcoding-based species detection can be accounted for by applying the multispecies occupancy modelling framework. We report a case study with the eDNA sample from an aquarium tank in which the detection probabilities of species in the two major steps of eDNA metabarcoding, filtration and PCR, across a range of PCR annealing temperatures, were examined. We also show that the results can be used to examine the efficiency of species detection under a given experimental design and setting, in terms of the efficiency of species detection, highlighting the usefulness of the multispecies site occupancy modelling framework to study the optimum conditions for molecular experiments.

## Introduction

Environmental DNA (eDNA) methods have been increasingly considered as useful tools in the investigation of the distribution of aquatic and terrestrial macroorganisms inhabiting various habitats^1–14^. Recently, high-throughput parallel DNA sequencing (HTS) has been applied in eDNA studies^1,3,14–21^ for simultaneous detection of multiple species from eDNA. This technique is called eDNA metabarcoding and is a rapid method of biodiversity assessment with DNA-based identification and HTS^14,16,17^. For example, Miya *et al*.^16^ developed MiFish primers (MiFish-U/E) to amplify a hypervariable region of the mitochondrial 12S rRNA gene, and tested the versatility of these PCR primers using eDNA from four aquaria with known species composition and that of natural seawater. They successfully detected eDNA from 232 fish species distributed across 70 families and 152 genera from the aquaria and the field, with a higher detection rate for species (>93%) in the aquaria. Such an eDNA metabarcoding technique has great potential as a useful tool for biodiversity assessment.

Species-detection via eDNA metabarcoding involves multiple sequential steps, such as field sampling, laboratory experiments, and bioinformatics^14,22^, each of which may be prone to species detection errors; false-negative and false-positive errors. False negative errors, failures to detect species that actually are present in the habitat, prevail in ecological field surveys^23^. Although the eDNA methodology may accomplish efficient detection of species^24^, it can still be subject to false negatives^25,24–27^. False negatives may occur even in laboratory experiments (e.g., PCR dropout)^27^. False positives also may occur even in laboratory experiments (e.g., DNA contamination from the environments and the other samples)^24^. Thus, the eDNA sampling approach can also suffer from false positive errors, because of contamination and/or errors in PCR or sequencing, which may result in the spurious detection of species^22,24,28,29^. It is clear that both false-positive and false-negative errors in the eDNA survey are critical, while the error rates have not yet been well investigated. Until now, ad hoc procedures have been proposed, such as not considering species detected in just a few PCR replicates. Despite these methodological issues, which are obviously critical to the assessment of biodiversity based on eDNA metabarcoding, they have not yet been well investigated, especially for laboratory experiments. Knowledge about detection error rates will contribute to determining an efficient sampling strategy and optimising the design and settings for laboratory experiments^25,26^. We should note that we do not evaluate false-positive rate in this study, due to the limited information for the modelling together with false-negative evaluation. We here focused on the false-negative rate in species detection by eDNA metabarcoding.

Rate of errors in species detection can be estimated within the site occupancy modelling framework. A site occupancy model^30^ is a hierarchical model that explains replicated detection/non-detection data of species across multiple sampling units (i.e., sites). It models species detection data conditional on the latent state of site occupancy (i.e., the existence of species within the site), thereby accounting for detection errors. Although the site occupancy model was originally developed to account for imperfect detection of species within a ‘site’ (e.g., a pond), it can also be applied to account for detection errors in laboratory experiments^31^ and has been proven to be useful for ecological surveys using eDNA^25,26,29^. An extension of the site occupancy model to the multispecies context, known as the multispecies site occupancy model^32,33^, can account for variation in detection probabilities among species. It therefore may have the potential to provide a powerful modelling framework for biodiversity assessments based on eDNA metabarcoding.

The objective of this study was to illustrate how the multispecies occupancy modelling framework can be used to evaluate probabilities of species detection in different steps of laboratory experiments for eDNA metabarcoding. As a case study, we present the results of eDNA metabarcoding for the fish community in a large aquarium with known fish species, where replicates were taken in the filtration and the PCR (1st PCR for library preparation) steps of the laboratory experiment. We estimated species-specific detection probabilities at these experimental steps by fitting a multispecies site occupancy model to the data, in which the dependence of the detection probability on the PCR annealing temperature was accounted for. In fact, the effect of the PCR annealing temperature has been shown to affect DNA metabarcoding and the use of inappropriate PCR conditions can also affect the final taxonomic assignment in metazoan metabarcoding analyses^34^. Given these estimates of detection probabilities, we show that the effectiveness of an experimental design and setting can be evaluated in terms of the efficiency of species detection.

## Methods

### Brief description of sampling and experimental design

**Figure 1:**
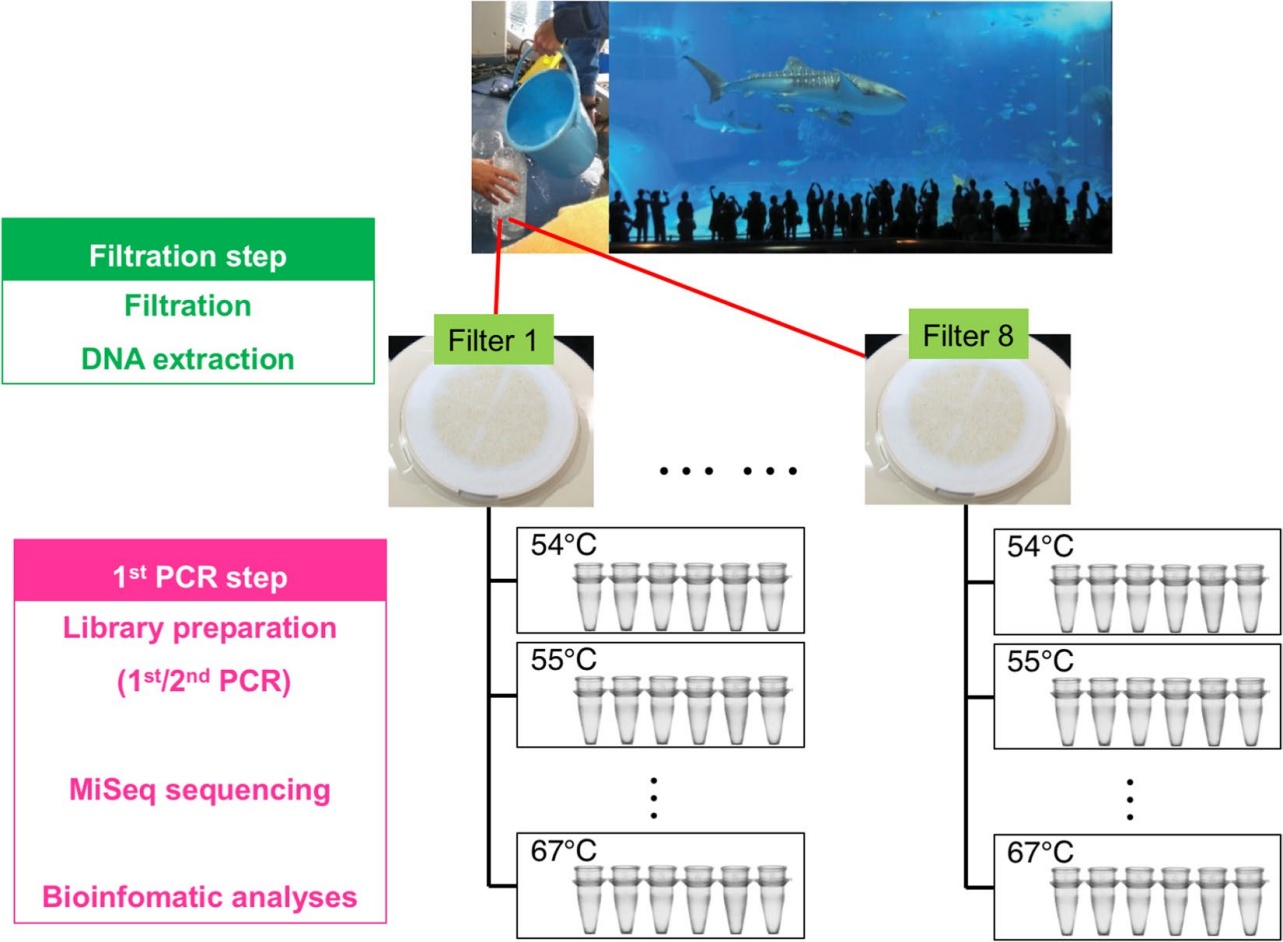
Sampling design for this study.

We developed an experimental design in which replicates were taken hierarchically to estimate detection probabilities at two experimental steps: (1) water filtration and (2) 1st PCR (Fig. 1). In addition, differences in the annealing temperatures at the 1st PCR were also considered (14 levels of temperature, 54–67 °C with one-degree intervals), totalling 672 PCR libraries (=8 filter replicates × 6 PCR replicates (including one non-template PCR blank) × 14 temperature levels) (Fig. 1). Here, we briefly describe the sampling and experimental procedure (see Appendix S1 for more details). First, we collected approximately 10 L of surface seawater from the Kuroshio tank (water volume = 7,500 m^3^) in the Okinawa Churaumi Aquarium, Okinawa, Japan (26º41′39″N, 127º52′41″E), where Miya *et al*.^16^ performed their eDNA metabarcoding study (see Appendix S1). The tank harbours taxonomically diverse fish species (ca. 63 species in the tank) from cartilaginous fish (sharks and rays) to bony fish. The sampled water was filtered through a 47-mm GF/F glass-fibre filter. In total, eight filter replicates were taken. Two litres of Milli-Q water was used as the equipment control to monitor contamination during filtering and subsequent DNA extraction. In the laboratory, we extracted the DNA from the filters using DNeasy blood and tissue kits (see Appendix S1). For MiSeq sequencing, we employed a two-step tailed PCR approach to construct the paired-end libraries; the 1st PCR was performed using two universal primer pairs (MiFish-U/E)^16^. Then, the 2nd PCR step and the sequence library preparation were performed (see Appendix S1). After sequencing, the Miseq-output data were prepared according to the bioinformatics pipeline process of Miya *et al*.^16^ (see Appendix S1 for the details and the software). Those sequences represented by >1 identical read and the remaining under-represented sequences (with <2 identical reads) were subjected to pairwise alignment. If the latter sequences observed from <2 reads showed ≥99% identity with one of the former reads, they were operationally considered identical (because of sequencing or PCR errors and/or actual nucleotide variations in the populations) and they were added to the >2 reads (see Appendix S1 for the details). All sequence data are available from the DDBJ/EMBL/NCBI Sequence Read Archives under the accession numbers DRA005190 and 005191.

### Data analysis

We developed a hierarchical model that could estimate the error rate for false negative detection of species that occurs at the filtration and 1st PCR steps. With the estimates of these error rates, we then considered the efficiency of some specific experimental designs and settings for detecting species eDNA in the sampled water. The following analysis was applied to data for fish species that were actually present in the aquarium; we omitted data for fish species that were absent from the tank to eliminate obvious false positive errors from the data. We could easily identify the false positives because the 12S rDNA sequences of all fish species in the aquarium were included in the database. For each species and sample, the MiSeq read data were reduced to detection/non-detection data. Species were treated as detected from a sample when the number of MiSeq reads of the sample was greater than that of the corresponding negative control. In this study, according to negative control, we fixed the detection threshold criteria. Also, we preliminary confirmed the same results from the data without using threshold criteria of negative control. This issue is discussed in many studies but there is little consensus about what criteria constitute a species detection. Thus, the setting of the detection threshold criteria would affect the results as suggested in the other study^35^. Because the number of reads was extremely low in a total of seven PCR replicates, probably due to a failure in the library preparation, data from these replicates were omitted. We analysed detection/non-detection observations of the 62 fish species detected by the MiFish sequencing (Table S1).

In the following analyses, false negative errors in species detection were formally accounted for, whereas false positive errors were not. Although analytical approaches that account for false positive errors may be applied in eDNA surveys^22,24,28,29^, the existence of false-positive errors complicates the problem of occupancy estimation considerably^36,37^, especially in a multispecies context. We note, however, that the unwanted effects of ignoring false positive errors should have been minimised in our study, because sampling from the aquarium enabled us to remove species that were absent from the analysed data, although we acknowledge that some unidentifiable false positives (e.g., cross-contamination) may yet remain in them.

A multispecies site occupancy model^32,33^ was fitted to the data to account for the false-negative detection error occurring at the two stages of the experiment (i.e., filtration and 1st PCR). Our model was a variant of the model described in Dorazio and Royle^32^, in which species-specific detection probabilities were estimated in accordance with their community-level distribution.

The term *x*_*ijk*_ denotes the number of detections of species *i* summed over *M*_*ijk*_ PCR replicates of temperature level *k* in filter *j* (for *i* = 1, ..., 62, *j* = 1, ..., 8, and *k* = 1, ..., 14, respectively). We considered the occurrence of eDNA of species *i* on filter *j*, which we denote by *z*_*ij*_, and modelled the data generating process as follows:

1 $${x}_{ijk} \sim {\rm{Binomial}}\,({M}_{ijk},{{\rm{\theta }}}_{ik}{z}_{ij})$$

2 $${z}_{ij} \sim {\rm{Bernoulli}}\,({{\rm{\psi }}}_{ij})$$

where *θ*_*ik*_ and *ψ*_*ij*_ are the conditional detection probabilities for species *i* for the 1st PCR of temperature level *k*, and the occurrence probability of species *i* for filter *j*, respectively. Note that in our formulation, filters and 1st PCR replicates correspond to ‘sites’ and ‘surveys’ in a conventional occupancy model, respectively. The occurrence of each species within the tank was not modelled here, because inference was restricted to species that were present in the tank.

We let *g*(*i*) be a function indicating the group of each species: *g*(*i*) = 1 if species *i* is a cartilaginous fish and *g*(*i*) = 2 if species *i* is a bony fish. Variation in occurrence/detection probability was decomposed as follows:

3 $${\rm{logit}}\,{{\rm{\psi }}}_{ij}={{\rm{\alpha }}}_{g(i)}+{{\rm{\alpha }}}_{g(i)j}^{({\rm{f}})}+{{\rm{\alpha }}}_{i}^{({\rm{s}})}+{{\rm{\alpha }}}_{ij}^{({\rm{fs}})}$$

4 $${\rm{logit}}\,{{\rm{\theta }}}_{ik}={{\rm{\beta }}}_{g(i)}+{{\rm{\beta }}}_{g(i)k}^{({\rm{t}})}+{{\rm{\beta }}}_{i}^{({\rm{s}})}+{{\rm{\beta }}}_{ik}^{({\rm{ts}})}$$

Here, α_*g*_, ${{\rm{\alpha }}}_{gj}^{({\rm{f}})}$, ${{\rm{\alpha }}}_{i}^{({\rm{s}})}$ and ${{\rm{\alpha }}}_{ij}^{({\rm{fs}})}$ are the group-specific intercepts (for *g* = 1, 2) of the filtration-level occurrence probability (on the logit scale), group-specific among-filter variation, species-specific variation, and interaction between filter and species, respectively. β_*g*_, ${{\rm{\beta }}}_{gk}^{({\rm{t}})}$, ${{\rm{\beta }}}_{i}^{({\rm{s}})}$, and ${{\rm{\beta }}}_{ik}^{({\rm{ts}})}$ are the group-specific intercept of the 1st PCR-level detection probability (on the logit scale), group-specific among-temperature variation, species-specific variation, and interaction between temperature and species, respectively. It was assumed that among-species variation in the error rate and interaction terms varied randomly, which respectively comes from a community-level prior normal distribution with mean 0:

5 $${{\rm{\alpha }}}_{i}^{({\rm{s}})} \sim {\rm{Normal}}(0,\,{\sigma }_{g(i)}^{({\rm{s}})2})$$

6 $${{\rm{\alpha }}}_{ij}^{({\rm{fs}})} \sim {\rm{Normal}}(0,\,{\sigma }_{g(i)}^{({\rm{fs}})2})$$

7 $${{\rm{\beta }}}_{i}^{({\rm{s}})} \sim {\rm{Normal}}(0,\,{\tau }_{g(i)}^{({\rm{s}})2})$$

8 $${{\rm{\beta }}}_{ik}^{({\rm{ts}})} \sim {\rm{Normal}}(0,\,{\tau }_{g(i)}^{({\rm{ts}})2})$$

where ${\sigma }_{g}^{({\rm{s}})2}$, ${\sigma }_{g}^{({\rm{fs}})2}$, ${\tau }_{g}^{({\rm{s}})2}$, and ${\tau }_{g}^{({\rm{ts}})2}$ are the group-specific variance parameters for each random effect.

The model was fitted in a Bayesian inference framework where vague prior distributions were specified for the unknown fixed parameters (i.e., α_*g*_, ${{\rm{\alpha }}}_{gj}^{({\rm{f}})}$, β_*g*_, ${{\rm{\beta }}}_{gk}^{({\rm{t}})}$, ${\sigma }_{g}^{({\rm{s}})2}$, ${\sigma }_{g}^{({\rm{fs}})2}$, ${\tau }_{g}^{({\rm{s}})2}$, and ${\tau }_{g}^{({\rm{ts}})2}$). We conducted the Markov chain Monte Carlo (MCMC) method in JAGS software version 4.2.0^38^ to obtain samples from the posterior distribution of parameters. Posterior samples were obtained from three independent chains of 100,000 iterations after a burn-in of 100,000 thinning at intervals of 100. The convergence of MCMC was affirmed by determining if the $\hat{R}$ statistic for each parameter of interest was less than 1.1.

Under the assumption that the statistical distributions of occurrence/detection probability were the same as in this experiment, an estimate for the proportion of the species detected for a given experimental design and settings could be derived for each group based on the parameter estimates of the model. We denote the expected proportion of the speies in group *g* detected in an experimental design with annealing temperature *k*, *J* filter replicates and *M* PCR replicates by *E*(*g*, *k*, *J*, *M*), which we termed the species detection efficiency. In Appendix S2, the derivation of this quantity is described in more detail.

## Results

### MiSeq sequencing

A MiSeq paired-end sequencing (2 × 150 bp) for the 672 PCR libraries yielded a total of 10,073,869 reads with 92.1% base-calls having Phred quality scores (Q) of ≥30.0 (excluding 8,617,531 reads from the PhiX spike-in control). This run was highly successful with the quality scores specified by Illumina ≥80% bases higher than Q30 at 2 × 150 bp (Illumina Publication No. 770-2011-001 as of May 27, 2014). After demultiplexing and pre-processing of the raw sequence data from MiSeq, the outputs were subjected to the BLAST searches for taxonomic assignment. In total, 6,634,131 reads were assigned to fish species with ≥97% identity to reference sequences in the custom database. Of these, 6,416,876 reads (96.7%) are identified as those fishes contained in the tank and the remaining 217,255 reads (3.3%) are derived from species absent from the tank. The rate of detection and the average of the log of the number of sequence reads is summarised in Figs S1 and S2, respectively.

### Occurrence/detection probabilities

**Figure 2:**
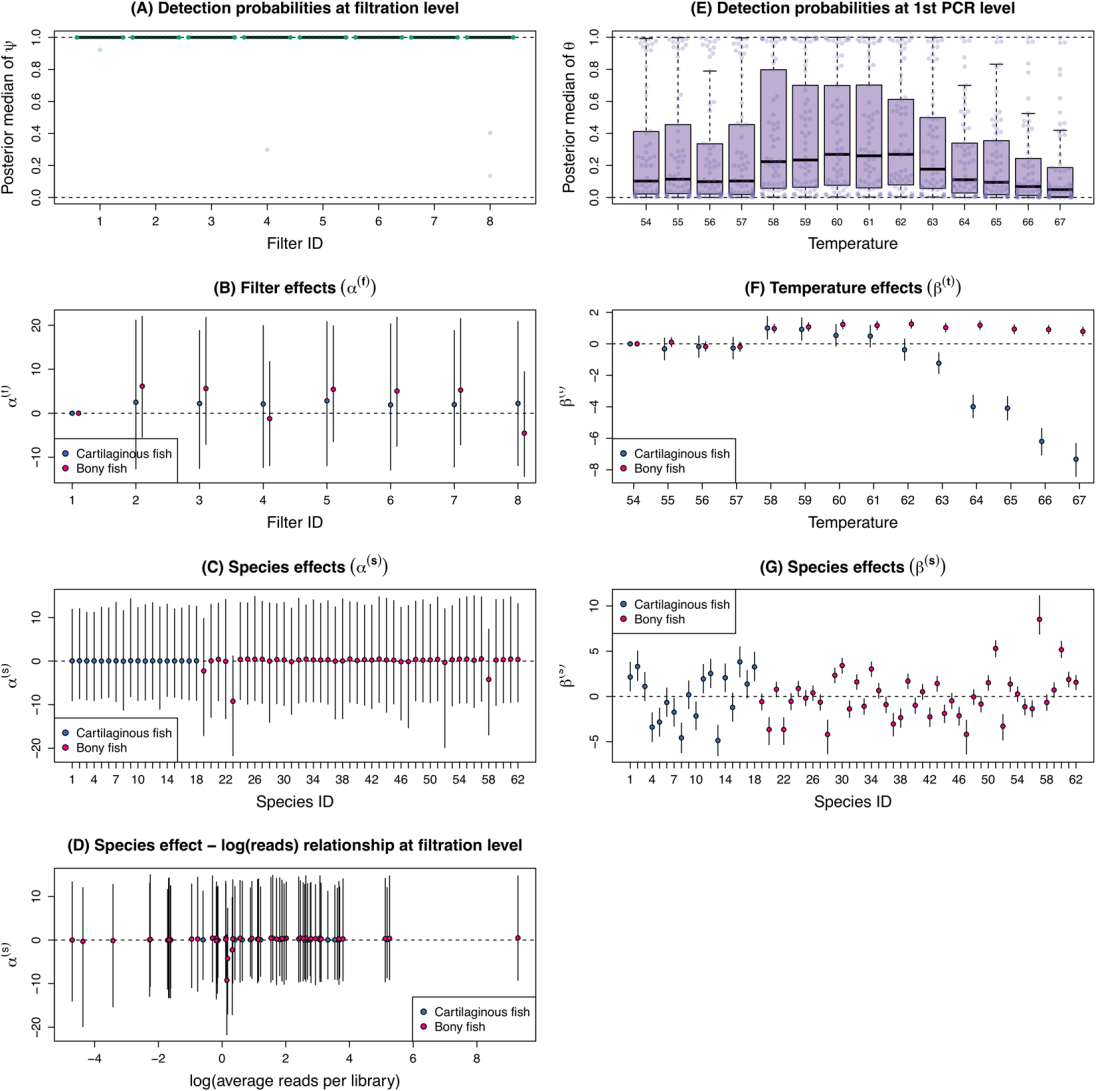
Result of the model fitting. (**A**) Filter-level occurrence probabilities; (**B**) filter replication effects on the occurrence probability; (**C**) species effects on the occurrence probability; (**D**) the relationship between the species effect on the occurrence probability and the log of reads; (**E**) 1st PCR level detection probabilities; (**F**) PCR annealing temperature effects on the detection probability; (**G**) species effects on the detection probability. Filled circles and error bars indicate medians and 95% credible intervals of the posterior distribution. For the species ID, refer to Appendix Table S1.

At the filtration level, the occurrence rate of species was uniformly very high. Most of the posterior medians of *ψ* were close to 1 (Fig. 2A), indicating that eDNA was successfully captured on each filter and very few species were missed at this level of the procedure. Reflecting this uniformly high level of the occurrence rate, significant variation was not found in the filter (${{\rm{\alpha }}}_{gj}^{({\rm{f}})}$), species (${{\rm{\alpha }}}_{i}^{({\rm{s}})}$), or their interaction (${{\rm{\alpha }}}_{ij}^{({\rm{fs}})}$) effects (Figs 2B,C, S3). We also found no obvious association between the species effect for the filtration-level occurrence probability (${{\rm{\alpha }}}_{i}^{({\rm{s}})}$) and the logarithms of the average number of reads (Fig. 2D), suggesting that, at filtration, eDNA was efficiently captured regardless of its concentration.

At the 1st PCR step, in contrast to the filtration step, the detection rate varied largely among species and temperature (Fig. 2E–G). Changes in detection probability along annealing temperatures were apparently different between two groups, bony and cartilaginous fishes. Estimates of temperature effects on the 1st PCR level detection probability (${{\rm{\beta }}}_{gk}^{({\rm{t}})}$) suggested that the detection rate of bony fish was consistently higher at temperature above 57 °C, whereas that of cartilaginous fish was maximised at 58–59 °C (Fig. 2F). In addition, the detection probability varied considerably among species (Figs 2G, S4). These results suggested that the detection probability at this step was associated with the concentration of eDNA in the samples.

### Species detection efficiency

**Figure 3:**
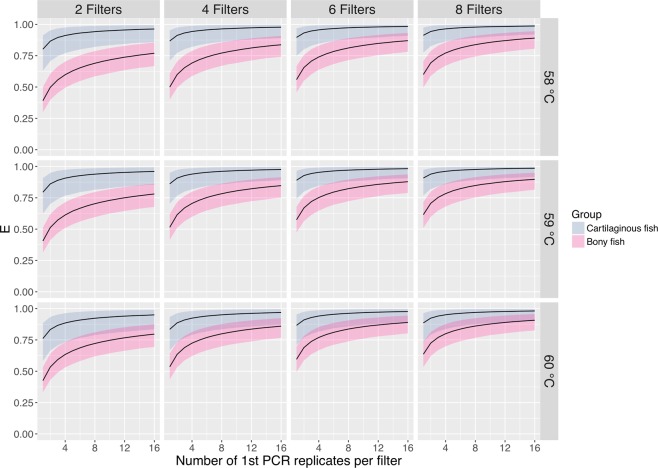
Species detection efficiency (*E*). Solid lines and coloured bands indicate medians and 95% credible intervals of the posterior distribution.

We evaluated the species detection efficiency with the parameter estimates for annealing temperatures of 58, 59 and 60 °C, filter replicates of 2, 4, 6 and 8, and PCR replicates of up to 16, which is shown in Fig. 3. We found that, at these levels of annealing temperatures, species of cartilaginous fish were captured more efficiently than those of bony fish. We noted that reflecting the high occurrence probability at the filtration level, the species detection efficiency depended entirely on the total number of the 1st PCR replicates in the experiment; hence, for a given set of *g*, *k*, *J*, and *M*, *E*(*g*, *k*, 0.5 *J*, 2 *M*) was virtually identical to *E*(*g*, *k*, *J*, *M*).

## Discussion

The multispecies occupancy modelling framework we employed enabled us to successfully quantify the probabilities of detecting DNA in a tank of aquarium at multiple experimental steps of the eDNA metabarcoding. Our example analysis showed consistently high occurrence rates at the filtration step, whereas relatively low and variable detection rates were seen in the 1st PCR step. A uniformly high occurrence probability at the filtration step suggests that a small number of filter replicates were sufficient to capture the eDNA of most species that were contained in the sampled water. On the other hand, low and variable detection probabilities at the 1st PCR step imply that increasing the number of PCR replicates would have improved the efficiency of species detection. This has been shown quantitatively by the species detection efficiency, which can be obtained with the estimated parameter values.

The results highlight the advantage of using the multispecies occupancy modelling framework for eDNA metabarcoding, which can help to determine the number of replicates at different experimental steps, as well as estimate the efficiency of species detection in a given experiment. In this case, we performed an experiment in an aquarium tank, thus, provided an practical example for the modelling and experimental frameworks to evaluate the efficiency of species detection in the eDNA metabarcoding.

PCR annealing temperature had significant effects on the detection rates. The DNA from two fish groups, bony and cartilaginous fishes, responded differently to PCR annealing temperature. For bony fish, the detection rate was consistently high at temperatures higher than 57 °C, whereas for cartilaginous fish, the detection rate had a positive hump-shaped relationship with PCR annealing temperature, which was maximised at 58–59 °C. This difference could have been caused by the different universal primers for bony and cartilaginous fishes because we used two universal primers (MiFish-U and E)^16^ in this study. From the results of Miya *et al*.^16^, MiFish-U can amplify DNA of most of bony fish species, and MiFish-E can amplify that of cartilaginous fish species. The primers, MiFish-U and E, have the same base length, but the *T*_m_ (melting temperature for PCR) of the primers were different; *T*_m_ of MiFish-U-F/R were 56.6 °C and 56.5 °C, respectively, and *T*_m_ of MiFish-E-F/R were 54.1 °C and 55.2 °C, respectively. The difference in *T*_m_, as well as that in the primer sequence, such as G/C content, might have influenced the responses observed for PCR annealing temperature. To maximise species detection using eDNA metabarcoding by MiFish primers, the suitable PCR temperature was 58–59 °C for both bony and cartilaginous fishes. The suitable temperature for PCR was 3–4 °C higher than the *T*_m_ values.

The design of perfectly matching universal primers for DNA metabarcoding can strongly influence sequencing performance^39–41^. Thus, the PCR bias associated with preferential amplification caused by primer mismatching is important for amplicon sequencing^40,41^. The MiFish universal primer almost perfectly matched the fish species in this study^16^. Other types of universal primers with greater mismatching for the targeted community might reduce the detection rate for the species and number of detected species by eDNA metabarcoding. In fact, using this method with MiFish primers, and sampling with four replicates produced higher detection rates than those obtained by Ficetola *et al*.^24^. In the current studies, using detection of the DNA reads by HTS, eDNA metabarcoding provide only qualitative data regarding the species^16,42^. However, some quantitative methods for metabarcoding using amplicon HTS have recently been developed^43,44^. For such quantitative evaluation, the PCR bias will be a critical issue^43,44^, because variation in PCR efficiency among species will influence the number of sequence reads by HTS. eDNA metabarcoding currently uses amplicon sequencing with universal PCR primers. However, HTS techniques have recently been developed for non-amplicon sequencing, for example, shotgun sequencing for HTS has been recently performed for metagenomes^1,45^. These new HTS technologies without PCR for library preparation could decrease the variability in the replicates for sequencing and increase the detection rate of species because the effect of PCR replication was relatively high in the detection rate for eDNA metabarcoding.

Our analysis showed consistently high detection rates at the filtration step, whereas relatively low and variable detection rates in the 1st PCR step. Our general recommendation is, therefore, to favour increasing the number of PCR replicates rather than filter replicates if the higher detection rates at the filtration step than the 1st PCR step. From our preliminary results in an aquarium, we encourage further research to quantify detection probabilities of eDNA metabarcoding. Although our results provided a useful guide for the allocation for replications in an eDNA metabarcoding analysis, it might not be quantitatively informative for other metabarcoding studies because of differences in field sampling conditions and experimental settings, which could result in differences in the detection probabilities at the experimental steps we examined.

Information on the probabilities of detecting species is critical to sampling and estimating biodiversity. The lack of such information can make a study more costly and time-consuming, while being less efficient. Using the our model framework, future researchers can quantify detection probabilities in eDNA metabarcoding. The differences in field sampling conditions and experimental settings may result in differences in the probabilities of detecting species eDNA. For example, detection probabilities could be different depending on the underlying population density. They may also be different if other universal primers for eDNA metabarcoding, such as ecoPrimer^46^ and Folmer’s COI primers^47^, were used. Other PCR conditions, including the PCR solution, number of cycles, and methods, might also influence the detection rate in the PCR step^40^. Adopting the multispecies occupancy modelling approach will be helpful to find an optimal sampling and experimental design for eDNA metabarcoding. Although the experimental and analytical approach we employed required the preliminary exclusion of false positive reads from the analysis because the hierarchical model we described only accounted for the false negative detection error, it would be applicable to eDNA metabarcoding data collected from the field, when a catalogue of species is available in the area where eDNA sampling was conducted.

## Supplementary information

Supplemental Materials

Supplemental Materials

## Data Availability

Sequencing data: All sequence data are available from the DDBJ/EMBL/NCBI Sequence Read Archives under the accession numbers DRA005190 and 005191.
