# Supplementary material for: Evaluation of detection probabilities at the water-filtering and initial PCR steps in environmental DNA metabarcoding using a multispecies site occupancy model
Source: Sci Rep. 2019 Mar 5;9:3581. doi: 10.1038/s41598-019-40233-1 (PMC6401178; doi:10.1038/s41598-019-40233-1)
Supplement: Supplementary file 1 — Supplemental Materials [file 41598_2019_40233_MOESM1_ESM.docx]

Evaluation of detection probabilities at the water-filtering and initial PCR steps in environmental DNA metabarcoding using a multispecies site occupancy model

Hideyuki Doi, Keiichi Fukaya, Shin-ichiro Oka, Keiichi Sato, Michio Kondoh, and Masaki Miya

Supplemental Materials

Appendix S1: Details of the experiments

Appendix S2: Derivation of species detection efficiency, *E*(*g*, *k*, *J*, *M*)

Fig S1: Heat map for the detection rate for each fish species and filter replicates along the PCR annealing temperature gradient. Species ID 1-18: Cartilaginous fish, 19-62: Bony fish; see Table S1 for species name.

Fig S2: Heat map for the mean log number of sequence reads for each fish species and filter replicates along the PCR annealing temperature gradient. Species ID 1-18: Cartilaginous fish, 19-62: Bony fish; see Table S1 for species name.

Fig S3: Filter-species interaction effects on the filtration-level occurrence probability. Error bars indicate 95% credible intervals. Species ID 1-18: Cartilaginous fish, 19-62: Bony fish; see Table S1 for species name.

Fig S4: Temperature-species interaction effects on the 1st PCR-level detection probability. Error bars indicate 95% credible intervals. Species ID 1-18: Cartilaginous fish, 19-62: Bony fish; see Table S1 for species name.

Table S1: List of species detected in the experiment.

**Appendix S1: Details of the experiments**

*Water sampling and filtering*

For water sampling from the aquarium, approximately 10 L of seawater was collected from the surface using three casts of an 8-L polyethylene bucket fastened to a 10-m rope. In total, we collected 30 L of seawater from three points to mitigate the heterogeneity of DNA in the water. The sampled water was stored in valve-equipped 10-L book bottles and immediately brought to the laboratory for filtering. For each filtering replicate, 2 L of well-mixed seawater from the 30-L samples was filtered onto 47-mm GF/F glass filters (GE Healthcare, Little Chalfont, UK). Two litres of Milli-Q water was used as the equipment control to monitor contamination during filtering and subsequent DNA extraction. Each filter was stored in -20°C before eDNA extraction. All sampling and filtering equipment was exposed to a 10% bleach solution for at least 30 min before use.

*DNA extraction*

DNA was extracted from the filters using a DNeasy Blood and Tissue Kit (Qiagen, Hilden, Germany) in combination with a spin column (EZ-10; Bio Basic, Markham, Ontario, Canada), following a protocol used in Miya et al. [1].

After removing the attached membrane from the spin column (EZ-10), the filter was tightly folded into a small cylindrical shape, and placed in the spin column. The spin column was centrifuged at 6,000 *g* for 1 min to remove redundant seawater before DNA extraction. The column was then placed in a new 2-mL tube and subjected to lysis using proteinase K. Before lysis, Milli-Q water (400 µL), proteinase K (20 µL), and buffer AL (180 µL) were mixed and the mixed solution was gently pipetted onto the folded filter in the spin column. Then, the column was placed on a 56°C preheated aluminium heat block for 30 min. The spin columns were covered with commercial aluminium foil and a clean blanket for effective incubation at the specified temperature. After the incubation, the spin column was centrifuged at 6,000 *g* for 1 min to collect the DNA. To increase DNA yield from the filter, 300 µL of sterilized TE buffer was gently pipetted onto the folded filter and the spin column was again centrifuged at 6,000 *g* for 1 min. The collected DNA was purified using the DNeasy Blood and Tissue Kit following the manufacture’s protocol.

*Library preparation and MiSeq sequencing*

The eDNA samples were used for multiplex PCR using two universal primer pairs for a hypervariable region of the mitochondrial 12S rRNA gene (163–185 bp) (MiFish-U/E; Miya et al. 2015). Prior to library preparation, work-space and equipment were sterilized, filtered pipet tips were used, and separation of pre- and post-PCR was carried out to safeguard against contamination. We also employed controls to monitor contamination, including PCR blanks for each experiment. For the paired-end sequencing on the MiSeq platform (Illumina, San Diego, CA), we employed a two-step tailed PCR approach to construct the paired-end libraries. The first-round PCR (1st PCR amplified the target region using primers 5´–ACACTCTTTCCCTACACGACGCTCTTCCGATCTNNNNNN + MiFish gene specific sequences –3´ (forward) and 5´–GTGACTGGAGTTCAGACGTGTGCTCTTCCGATCTNNNNNN + MiFish gene specific sequences –3´ (reverse). The first 33 and 34 nucleotides (nt) were partially used for primer binding sites for sequencing and the following six random hexamers (N) were used to enhance cluster separation on the flowcells during initial base call calibrations on the MiSeq platform.

The 1st PCR was conducted with 35 cycles of a 12-µL reaction volume containing 6.0 µL 2× KAPA HiFi HotStart ReadyMix (KAPA Biosystems, Wilmington, MA), 0.7 µL of each primer (5 µM), 2.6 µL sterile distilled H_2_O, and 2.0 µL template according to Miya et al. (2015). The thermal cycle profile after an initial 3-min denaturation at 95°C was as follows: denaturation at 98°C for 20 s; annealing at various temperatures from 54 to 67°C in 1°C intervals for 15 s (Fig. 1); and extension at 72°C for 15 s, with the final extension at the same temperature for 5 min.

The second-round PCR (2nd PCR) used the 1st-PCR products as a template and amplified the region using primers 5´–AATGATACGGCGACCACCGAGATCTACAXXXXXXXXACACTCTTTCCCTACACGACGCTCTTCCGATCT –3´ (forward) and 5´– CAAGCAGAAGACGGCATACGAGATXXXXXXXXGTGACTGGAGTTCAGACGTGTGCTCTTCCGATCT –3´ (reverse). The octo-X segments represented dual-index sequences (44 unique indices in total). The 5´ end sequences were adapters that allowed the final product to bind or hybridize to short oligos on the surface of the Illumina flowcell. The 3´ end sequences were priming sites for the MiSeq sequencing. The 1st PCR product was 10-times diluted using Milli-Q water for a template for the 2nd PCR. The 2nd PCR was conducted with 12 cycles of a 12-µL reaction volume containing 6.0 µL 2× KAPA HiFi HotStart ReadyMix, 0.7 µL each primer (5 µM), 3.6 µL sterile distilled H_2_O, and 1.0 µL template. Different combinations of indices (chosen from M501–532 for forward primers and A/D701–712 for reverse primers) were used for different templates for a massively parallel sequencing using the MiSeq platform. The thermal cycle profile after an initial 3-min denaturation at 95°C was as follows: denaturation at 98°C for 20 s; annealing and extension combined at 72°C (shuttle PCR) for 15 s, with the final extension at the same temperature for 5 min.

The indexed 2nd PCR products were pooled in equal volumes and the pooled libraries (total 100 µL) were subjected to agarose gel electrophoresis using 2% L03 (Takara, Otsu, Japan). The target size of the libraries (ca. 370 bp) was excised from the gel and purified using a MinElute Gel Extraction kit (Qiagen, Hilden, Germany) with an elution volume of 12 µL. The library concentration was estimated by a Qubit dsDNA HS assay kit and a Qubit 2.0 fluorometer (Life Technologies, Carlsbad, CA). Double-stranded DNA concentration of the pooled library was adjusted to 4 nM (assuming one base pair equals 660 g/mol) using Milli-Q water and 5 µL of the 4-nM library was denatured with 5 µL of fresh 0.1 N NaOH. Including the HT1 buffer (provided by the Illumina MiSeq v2 Reagent kit for 2 × 150 bp PE), the denatured library (10 µL; 2 nM) was diluted to the final concentration of 12 pM for sequencing on the MiSeq platform. A 240-µL volume of PhiX DNA spike-in control (12 pM) was added to 360 µL of the diluted library to improve data quality of low diversity samples, such as single PCR amplicons, used in this study.

*Bioinformatic analysis for MiSeq sequencing*

The processing for Miseq-output data was conducted according to the pipeline process of Miya et al. [1]. The overall quality of the MiSeq reads was evaluated by the programs FastQC (http://www.bioinformatics.babraham.ac.uk/projects/fastqc/) and SUGAR [2]. After confirming a lack of technical errors in the MiSeq sequencing, low-quality tails were trimmed from each read using DynamicTrim.pl from the SolexaQA software package [3] with a cutoff threshold set at a Phred score of 10 [4]. The tail-trimmed paired-end reads (Reads 1 and 2) were assembled using the software FLASH [5] with a minimum overlap of 10 bp. The assembled reads were further filtered by custom Perl scripts to remove reads with either ambiguous sites (Ns) or those exhibiting unusual lengths with reference to the expected size of the PCR amplicons (297 ± 25 bp). Finally, the software TagCleaner [6] was used to remove primer sequences with a maximum of three-base mismatches and to transform the FASTQ [7] format into FASTA. The pre-processed reads from the above custom pipeline were dereplicated using a “derep_fulllength” command in UCLUST [8], with the number of identical reads added to the header line of the FASTA formatted data file. Those sequences represented by > 1 identical reads were subjected to the downstream analyses and the remaining under-represented sequences (with < 2 identical reads) were subjected to pairwise alignment using a “usearch_global” command in UCLUST. If the latter sequences observed from less than 2 reads showed ≥ 99% identity with one of the former reads (1 or 2 nucleotide differences), they were operationally considered identical (because of sequencing or PCR errors and/or actual nucleotide variations in the populations) and they were added to the > 2 reads. The processed reads were subjected to local BLASTN searches [9] against a custom-made database. The latter was generated by downloading all entire and partial fish mitogenome sequences deposited in MitoFish [10] and entire mitogenome sequences from tetrapods deposited in NCBI Organelle Genome Resources (http://www.ncbi.nlm.nih.gov/genomes/OrganelleResource.cgi?taxid=32523) to cover tetrapods occurring in aquatic environments. In addition, the custom database was supplemented by assembling new sequences in MM’s lab, as of April 12, 2016. The top BLAST hit with a sequence identity of ≥ 97% and *E*-value threshold of 10^-5^ was applied to species assignments of each representative sequence. If a query sequence was aligned to the top BLAST hit sequence with an alignment length of 150 bp with one mismatch present, the ratio was calculated as 150/(1 + 1). A value of one was added to the denominator to avoid zero-divisors. This ratio was calculated for the top and second BLAST hit species, and a LOD (log of odds ratio) score between these ratios was used as a comparable indicator of the species assignment.

**Appendix S2: Derivation of species detection efficiency, *E*(*g*, *k*, *J*, *M*).**

We denoted a vector of the observations of a species, that belongs to group *g* (*g* = 1, if the species is a cartilaginous fish and *g* = 2 if the species is a bony fish; see the main text) from *J* filter samples by $\boldsymbol{x}=(x_{1},x_{2},\ldots,x_{J})$. The model we considered gives the marginal probability of the observation vector $\boldsymbol{x}$, under an experimental system with the annealing temperature *k* with *J* filter replicates and *M* PCR replicates for each filter. It is described as

$$q\left( \boldsymbol{x} | \boldsymbol{\xi}_{g},k,J,M \right)=\int\int\int\int\prod_{j=1}^{J} p\left( x_{j} | \psi_{j},\theta_{k},M \right)p\left( \alpha^{\left( s \right)} | \sigma_{g}^{\left( s \right)2} \right)p\left( \boldsymbol{\alpha}^{\left( \mathrm{fs} \right)} | \sigma_{g}^{\left( \mathrm{fs} \right)2} \right)p\left( \beta^{\left( s \right)} | \tau_{g}^{\left( s \right)2} \right)p\left( \beta^{\left( \mathrm{ts} \right)} | \tau_{g}^{\left( \mathrm{ts} \right)2} \right)$$

$$d\alpha^{(s)}d\boldsymbol{\alpha}^{(\mathrm{fs})}d\beta^{(s)}d\beta^{(\mathrm{fs})}$$

where $\boldsymbol{\xi}_{g}=(\alpha_{g},\alpha_{g1}^{(f)},{\alpha_{g2}^{(f)},\ldots,\alpha}_{gJ}^{(f)},\beta_{g},\beta_{gk}^{(t)},\sigma_{g}^{\left( s \right)2},\sigma_{g}^{(\mathrm{fs})2},\tau_{g}^{\left( s \right)2},\tau_{g}^{\left( ts \right)2})$, $\boldsymbol{\alpha}^{(\text{fs})}=(\alpha_{1}^{(\text{fs})},\alpha_{2}^{(\text{fs})},\ldots,\alpha_{J}^{(\text{fs})})$, and *p*(*a* | *b*) is the probability (density) distribution of *a* that is conditional on *b*, which was specified in the model. The integral can be evaluated numerically using Monte Carlo integration (see below). The complement of $q(\boldsymbol{x}=\mathbf{0}|\boldsymbol{\xi}_{g},k,J,M)$ gives the marginal probability of the eDNA from a randomly selected species in group *g* existing in the sampled water is detected in an experimental system (*k*, *J*, *M*):

$E\left( g,k, J, M \right)=1-q(\boldsymbol{x}=\mathbf{0}|\boldsymbol{\xi}_{g},k,J,M)$.

This quantity represents the expected proportion of the species that is detected and can also be used to estimate the number of species included in the sampled water [11].

For a given $\boldsymbol{\xi}_{g}$, $q(\boldsymbol{x}=\mathbf{0}|\boldsymbol{\xi}_{g},k,J,M)$ can be evaluated numerically by the following procedure (Monte Carlo integration):

1. Draw a sample of random effect parameters from the following distributions:
    $\alpha^{(\text{s})}$ ~ Normal(0, $\sigma_{g}^{\left( \text{s} \right)2}$),

$\alpha_{j}^{(\text{fs})}$ ~ Normal(0, $\sigma_{g}^{\left( \text{fs} \right)2}$) (for *j* in 1, …, *J*),

$\beta^{(\text{s})}$ ~ Normal(0, $\tau_{g}^{\left( \text{s} \right)2}$),

$\beta^{(\text{ts})}$ ~ Normal(0, $\tau_{g}^{\left( \text{ts} \right)2}$).

2. Calculate the conditional probability $p(\boldsymbol{x}=0|\boldsymbol{\psi},\theta_{k},M)$ as follows:

$p\left( \boldsymbol{x}=\mathbf{0} | \boldsymbol{\psi},\theta_{k},M \right)=\prod_{j=1}^{J} p(x_{j}=0|\psi_{j},\theta_{k},M)=\prod_{j=1}^{J} [\psi_{j}{(1-\theta_{k})}^{M}+(1-\psi_{j})]$,

where $\psi_{j}=\text{logit}^{-1}(\alpha_{g}+\alpha_{gj}^{(\text{f})}+\alpha^{(\text{s})}+\alpha_{j}^{(\text{fs})})$ and $\theta_{k}=\text{logit}^{-1}(\beta_{g}+\beta_{gk}^{(\text{t})}+\beta^{(\text{s})}+\beta^{(\text{ts})})$.

1. Repeat steps 1 and 2 many (e.g., 10,000) times. $q(\boldsymbol{x}=\boldsymbol{0}|\boldsymbol{\xi}_{g},k,J,M)$ is then approximated by the average of $p(\boldsymbol{x}=0|\boldsymbol{\psi},\theta_{k},M)$.

In this study, we estimated posterior distribution of *E*(*g*, *k*, *J*, *M*), by applying the above procedure for each posterior sample of parameters. An R script to obtain the posterior distribution of *E*(*g*, *k*, *J*, *M*) is supplied as a supplementary material. Note that to obtain *E*(*g*, *k*, *J*, *M*), we set $\alpha_{gj}^{(\text{f})}$= 0 for all *g* and *j* values, because no significant filter effect was found in our analysis (Figure 2B).

References

1 Miya, M. et al. MiFish, a set of universal PCR primers for metabarcoding environmental DNA from fishes: Detection of more than 230 subtropical marine species. *Royal Soc. Open Sci.* **2,** 150088 (2015).

2 Sato, Y. et al. SUGAR: graphical user interface-based data refiner for high-throughput DNA sequencing. *BMC Genomics* **15,** 664 (2014).

3 Cox, M. P., Peterson, D. A. & Biggs, P. J. SolexaQA: At-a-glance quality assessment of Illumina second-generation sequencing data. *BMC Bioinformatics*, **11,** 485 (2010).

4 Ewing, B., Hillier, L., Wendl, M. C. & Green, P. Base-calling of automated sequencer traces using Phred. I. Accuracy assessment. *Genome Res.* **8,** 175–185 (1998).

5 Magoč, T. & Salzberg, S. L. FLASH: fast length adjustment of short reads to improve genome assemblies. *Bioinformatics* **27,** 2957–2963 (2011).

6 Schmieder, R., Lim, Y. W., Rohwer, F., & Edwards, R. TagCleaner: Identification and removal of tag sequences from genomic and metagenomic datasets. *BMC* *Bioinformatics* **11,** 341 (2010).

7 Cock, P. J., Fields, C. J., Goto, N., Heuer, M. L. & Rice, P. M. The Sanger FASTQ file format for sequences with quality scores, and the Solexa/Illumina FASTQ variants. *Nucleic Acids Res*. **38,** 1767–1771 (2010).

8 Edgar, R. C. Search and clustering orders of magnitude faster than BLAST. *Bioinformatics* **26,** 2460–2461 (2010).

9 Camacho, C. et al. BLAST+: architecture and applications. *BMC Bioinformatics* **10,** 421 (2009).

10 Iwasaki, W. et al. MitoFish and MitoAnnotator: A mitochondrial genome database of fish with an accurate and automatic annotation pipeline. *Mol. Biol. Evol.* **30,** 2531–2540 (2013).

11 Dorazio, R. M. & Royle, J. A. Estimating size and composition of biological communities by modeling the occurrence of species. *J. Ame. Stat. Assoc.* **100,** 389–398 (2005).

Figure S1

Figure S2

Figure S3

Figure S4

Table S1

| Species ID | Scientific name | Fish group |
| --- | --- | --- |
| 1 | *Nebrius ferrugineus* | Cartilaginous fish |
| 2 | *Rhincodon typus* | Cartilaginous fish |
| 3 | *Stegostoma fasciatum* | Cartilaginous fish |
| 4 | *Carcharhinus leucas* | Cartilaginous fish |
| 5 | *Carcharhinus plumbeus* | Cartilaginous fish |
| 6 | *Galeocerdo cuvier* | Cartilaginous fish |
| 7 | *Negaprion acutidens* | Cartilaginous fish |
| 8 | *Sphyrna lewini* | Cartilaginous fish |
| 9 | *Rhynchobatus djiddensis* | Cartilaginous fish |
| 10 | *Rhina ancylostoma* | Cartilaginous fish |
| 11 | *Dasyatis* sp. | Cartilaginous fish |
| 12 | *Dasyatis ushiei* | Cartilaginous fish |
| 13 | *Himantura microphthalma* | Cartilaginous fish |
| 14 | *Himantura uarnak* | Cartilaginous fish |
| 15 | *Urogymnus asperrimus* | Cartilaginous fish |
| 16 | *Aetobatus narinari* | Cartilaginous fish |
| 17 | *Manta birostris* | Cartilaginous fish |
| 18 | *Rhinoptera javanica* | Cartilaginous fish |
| 19 | *Elops hawaiensis* | Bony fish |
| 20 | *Chanos chanos* | Bony fish |
| 21 | *Epinephelus lanceolatus* | Bony fish |
| 22 | *Epinephelus moara* | Bony fish |
| 23 | *Epinephelus tukula* | Bony fish |
| 24 | *Coryphaena hippurus* | Bony fish |
| 25 | *Rachycentron canadum* | Bony fish |
| 26 | *Echeneis naucrates* | Bony fish |
| 27 | *Alectis indica* | Bony fish |
| 28 | *Carangichthys dinema* (Uraspis) | Bony fish |
| 29 | *Caranx ignobilis* | Bony fish |
| 30 | *Caranx melampygus* | Bony fish |
| 31 | *Caranx papuensis* | Bony fish |
| 32 | *Caranx sexfasciatus* | Bony fish |
| 33 | *Decapterus macarellus* | Bony fish |
| 34 | *Elagatis bipinnulata* | Bony fish |
| 35 | *Gnathanodon speciosus* | Bony fish |
| 36 | *Naucrates ductor* | Bony fish |
| 37 | *Pseudocaranx dentex* | Bony fish |
| 38 | *Scomberoides lysan* | Bony fish |
| 39 | *Selar crumenophthalmus* | Bony fish |
| 40 | *Selaroides leptolepis* (Atule) | Bony fish |
| 41 | *Seriola dumerili* | Bony fish |
| 42 | *Seriola rivoliana* (Naucrates) | Bony fish |
| 43 | *Trachinotus blochii* | Bony fish |
| 44 | *Uraspis helvola* | Bony fish |
| 45 | *Aprion virescens* | Bony fish |
| 46 | *Lutjanus bohar* | Bony fish |
| 47 | *Pristipomoides argyrogrammicus* | Bony fish |
| 48 | *Caesio caerulaurea* | Bony fish |
| 49 | *Caesio cuning* | Bony fish |
| 50 | *Caesio teres* | Bony fish |
| 51 | *Pterocaesio chrysozona* | Bony fish |
| 52 | *Pterocaesio digramma* | Bony fish |
| 53 | *Pterocaesio tile* | Bony fish |
| 54 | *Lethrinus nebulosus* | Bony fish |
| 55 | *Lethrinus olivaceus* | Bony fish |
| 56 | *Auxis thazard* | Bony fish |
| 57 | *Euthynnus affinis* | Bony fish |
| 58 | *Gymnosarda unicolor* | Bony fish |
| 59 | *Katsuwonus pelamis* | Bony fish |
| 60 | *Rastrelliger kanagurta* | Bony fish |
| 61 | *Thunnus albacares* | Bony fish |
| 62 | *Thunnus orientalis* | Bony fish |
